# Space to play: identifying children's sites in the Pleistocene archaeological record

**DOI:** 10.1017/ehs.2020.29

**Published:** 2020-06-01

**Authors:** Michelle C. Langley

**Affiliations:** Australian Research Centre for Human Evolution, Environmental Futures Research Institute, Griffith University, Australia; and Forensics and Archaeology, School of Environment and Science, Griffith University, Australia

**Keywords:** Childhood, adolescence, Palaeolithic, children's places, secret spaces

## Abstract

Identifying the residues of children's activities in deep time contexts is essential if we are to build a comprehensive understanding of human cognitive and cultural development. Despite the importance of such data to human evolution studies, however, archaeologists have only recently begun to look for prehistoric children's material culture, and the identification of children's spaces is completely absent for deep time contexts. This paper draws together sociological and historical data regarding the universal need of *Homo sapiens* children for ‘secret’ places – places away from parental control. These spaces are important for the behavioural development of children and are universal in modern contexts. This paper demonstrates that these features can be identified in prehistoric archaeological records – and as such – researchers will have new datasets with which to interrogate the role of children in the development of their respective societies.

## Graphical abstract

**Figure:**
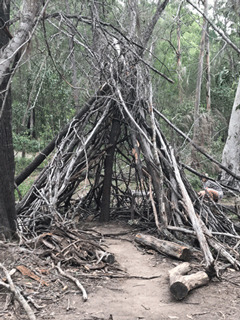

**Media summary:** Sociological and psychological data is applied to the archaeological record to find children's places in the deep past.

## Introduction

Unravelling and understanding the processes behind behavioural evolution, cultural development and technological innovation has always been central to archaeological research. For decades researchers have debated the merits and impact of changing population sizes and densities (e.g. Richerson et al., 2009; Shennan, 2001), climate change (e.g. Barton et al., 2007; Ziegler et al., 2013), mobility and social interconnectedness (e.g. Grove, 2016; Lycett & Norton, 2010), and diet (e.g. Parkington, 2010) among other factors on our cognitive and cultural capabilities. One agent yet to be seriously considered in these discussions, however, is the role of children in technological and cultural innovation throughout the deep past.

Childhood archaeology has established itself over the past 30 (but particularly 10) years within the wider discipline (Baxter, 2005a; Lillehammer, 1989; Sofaer Derevenski, 2000), although study of children outside of burial contexts or the transmission of lithic technological know-how in prehistoric – particularly Pleistocene – contexts remains peripheral (but see Langley, 2017; Langley & Litster, 2018; Langley et al., 2020; Nowell, 2015, 2016; Riede et al., 2017). Having said this, steps forward have been taken over the past few years, with researchers focusing on how we can go about identifying children's material culture items within the assemblages recovered (Langley, 2017; Langley & Litster, 2018; Pfeifer, 2015). With this aspect gaining some level of consensus and understanding in the literature, it is now time for researchers to turn their attention to identifying children's spaces – for it is these records that will allow us the greatest insights into what roles children played in changes in their community's technologies and culture. Here, a methodology for identifying spaces created and utilised by *Homo sapiens* children in the deep past is outlined and tested against a Palaeolithic record.

## Children's places and ‘secret’ spaces

**Figure 1.**
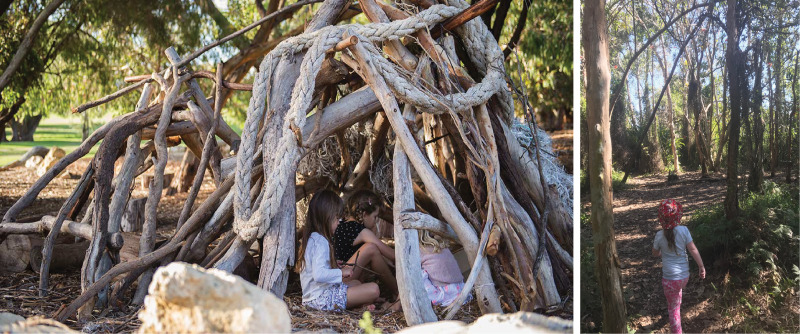
Children's cubby houses and activity areas in present-day suburban Australia. Left: girls playing in a cubby house at Russell Brown Adventure Park, Western Australia (photograph: Amanda Miller for NatureBasedPlay.com.au, 2017). Right: author's daughter entering a child-produced play area in the bush, Calamvale, Queensland (photograph: M. Langley, 2018).

The existence and importance of children's ‘special’ or ‘secret’ spaces have been studied extensively by sociologists, psychologists and educators. This behaviour – the creation of a place visited frequently by the child (and often chosen siblings and/or friends) – is recognised as a universal feature of childhood (Baxter, 2005a; Moore, 2015; Sobel, 1990, 1993; Sturm, 2008). These secret spaces are known by a variety of terms including ‘cubby houses’, ‘playhouses’, ‘dens’, ‘hideouts’, ‘forts’, and ‘bush houses’ (Figure 1).

The universality of this behaviour may stem from ‘an innate evolutionary drive for children to create hiding spaces or places – a vestigial survival strategy’ (Elliot, 2010, p. 61) where young children, just beginning to seperate from their parents, will actively seek out places of refuge (Heerwagen & Orians, 2002). Along similar lines, Jack (2010, p. 756) has suggested that there is a primordial ‘urge’ to seek out ‘your own place’. However, Nabhan and Trimble (1994) and more recently Cloke and Jones (2005) contend that children's motivation to create secret places is more than just a primitive survival urge, instead suggesting that it is more about creating a place seperate from the adult-controlled world. Indeed, children themselves have described to researchers that their motivation for establishing secret spaces is to ‘find a location that adults didn't know about’ (Roe, 2007, p. 477) and for ‘hiding from grown-ups’ (Moore, 2015, p. 27). Furthermore, it was communicated to Moore (2015, p. 21) by her preschool-aged informants that ‘only children can make secret places and that adults are not capable of doing so on children's behalf’. Thus, while it appears that the primary motivation for the creation of secret places is to escape the view of adults, this behaviour still involves potential evolutionary aspects as these places support the psychological and cognitive development of children, as well as providing a private place for them to practice social and practical skills (Elliot, 2010; Kirkby, 1989; Kriesberg, 1999; Singer & Singer, 1990; White, 2008; Wilson, 1997).

‘Secret’ spaces share a number of common characteristics. First, each space is found and/or constructed by a child on their own. Often children will take up space unused or abandoned by adults (Baxter, 2005a), and can actually be very close to home or in very public locations (Lim & Barton, 2010; Sobel, 1993). Rasmussen (2004), for example, found that the majority of important places nominated by children were ‘pieces of ground … trees … and corners’ (p. 161), places which were ‘often unnoticed by adults’ (p. 155) or considered to be ‘examples of disorder, mess, destruction and prohibited behaviour’ (p. 162) rather than a cherished place. The most important factor of these spaces is that they are ‘owned’ by the child/children involved. Once designated as ‘secret’, knowledge surrounding the existence and location of the place is carefully guarded by the child/children, and once they are inside their place it is important that they are not seen (Moore, 2015; Sobel, 1993).

Young children can develop strong bonds to places they experience (Jack, 2010; Lim & Barton, 2010; Measham, 2006), and these created and owned places fall into such a category. Indeed, these places are only attributed as ‘secret’ when special meaning has been given to the place by the child and they therefore possess it (Rasmussen, 2004). In this secret place ‘a child feels both connected (internally to place and self) and disconnected (externally from rules, adults and daily routines) and the child retains the power to observe the world without being observed by the world’ (Sturm, 2008, p. 47). Here, ‘a feeling of calm and repose comes over children when they are in their special places. There is often a reflective or meditatively quiet aspect to being in these places’ (Sobel, 1993, pp. 95–96). One of Moore's (2015, p. 27) informants related to her that a secret place is ‘very peace and quiet because it has a little hole in it so you can sneak in and no one can see you … not a bit … Inventions need very, very quiet places … and that's why they do it [make secret places], to get in very quiet places’. That is, that the secret place enables imaginative thinking and ‘illustrates children's capacities to construct places to provide time alone to make meanings of their world’ (Moore, 2015, p. 27).

Interestingly, there appear to be some age-based differences in the construction and meaning of children's secret places. In his cross-cultural investigation, Sobel (1993) found that the creation and use of ‘secret spaces’ by children in the UK and in the Caribbean became significant around the age of 6 or 7, and reached the height of importance for children at around the age of 8–11. His observations led him to conclude that two major categories of ‘dens’ existed: (a) those that were primarily individual, private places; and (b) those that were for a set membership of children. He found that the shared places were important throughout the later middle childhood (ages 8–11), but that the private dens became increasingly important from children around ages 10 and 11. Similarly, Silvey & MacKeith (1988) found that 74% of their survey respondents began creating paracosms between the ages of 7 and 12. For the second type of ‘den’ (that used by several children), Sobel found that children up to about the age of 8 often described mixed girl/boy play – games often imitating adult role-play such as ‘families’ and ‘shopping’ – at these locations. From 8 to 11, however, he reports that this pattern changes, with girls becoming more focused on working on the interior details of their playhouse, while boys concentrated more on building complex structures with walls and roofs. Another factor that he noticed was that the bringing of food to the secret place was important to the children – that it ‘consecrates the specialness of the place’ (Sobel, 1993, p. 46). Such physical aspects may be useful to archaeologists in determining the age of children creating identified activity areas.

While I have referred to Sobel's cross-cultural work between the UK and the Caribbean above, it is important to note that children across the globe and in various cultural contexts have been observed creating playhouses. Indeed, reviews of ethnographies for modern hunter–gatherer communities find that the construction of cubbies is a prominent feature of their children's play, being reported for peoples across the Australian continent, Central America, South America, North America, Africa, Southeast Asia, and islands within the Pacific Ocean (see Langley & Lister, 2018 for summary). Descriptions are usually confined to brief mentions of this behaviour, however, such as short statements along the lines of ‘they make play houses of the same kind as those in which they live and play at keeping house’ (Thomson 1958, p. 91 in reference to Cape York) and children get ‘stiff necks from playing all day in miniature *mia-mias* that they build for themselves’ (Kennedy & Donaldson 1982, p. 8 in reference to Ngamba children). More focused observation of this behaviour globally is an area worthy of academic attention in the future.

## Children's use of space in childhood archaeology

There has been very limited discussion of children's use of space and how to go about the identification of children's places in the archaeological literature. Most text has been preoccupied with suggesting that children have a randomising and/or distorting effect on artefact distributions (primarily Hammond & Hammond, 1981; but also mentioned in Bonnichsen, 1973; Deal, 1985; Hayden & Cannon, 1983; Schiffer, 1989; Stevenson, 1990; Watson, 1979; Wilk & Schiffer, 1979), although more recent analyses have disputed this assertion by pointing out the inappropriateness of the utilised data (Baxter, 2005b). Similarly, assertions that children's activity areas tend to occur in areas peripheral to adult activity have been challenged (Baxter, 2005b), and difficulties in recognising children's play in the archaeological record identified (Baxter, 2005b; Langley & Litster, 2018). Having said this, it is also recognised that, because children use secret places, this behaviour should produce successive episodes of child activity at a specific location creating a concentration of material items and that ‘these spaces should have a high degree of occurrence in archaeological sites and should be recognisable in the archaeological record’ (Baxter, 2005b, p. 72). Thus far, however, studies which have specifically focused on identifying children's spaces in archaeological contexts have thus far been limited to only two studies, both concentrating on historic period sites in the USA (i.e. Baxter, 2000, 2005a, b; Wilkie, 1994).

Drawing together this research just outlined, a number of characteristics which may identify the archaeological recording of a child's secret place can be proposed:

1. a location ‘out of sight’ of adults (Cloke & Jones, 2005; Moore, 2015; Nabhan & Trimble, 1994; Roe, 2007);
2. small site size (Baxter, 2005b; Moore, 2015; Sobel, 1993);
3. mimicry of the main housing type utilised by the child's community (Langley & Litster, 2018; Sobel, 1993);
4. ephemeral in nature (Moore, 2015; Rasmussen, 2004; Sobel, 1993);
5. A structure may be present – but not necessarily (age dependant) (Sobel, 1993);
6. low-value items and refuse may be present as these materials are often collected by children (Baxter, 2005b);
7. children's material culture (Baxter, 2005b; Langley & Litster, 2018; Sobel, 1993; Wilkie, 1994); and
8. possible food refuse (Sobel, 1993).

To explore these characteristics further, a case study from Upper Paleolithic Western Europe will now be presented.

## A case study: Étiolles (Essonne, France)

**Figure 2.**
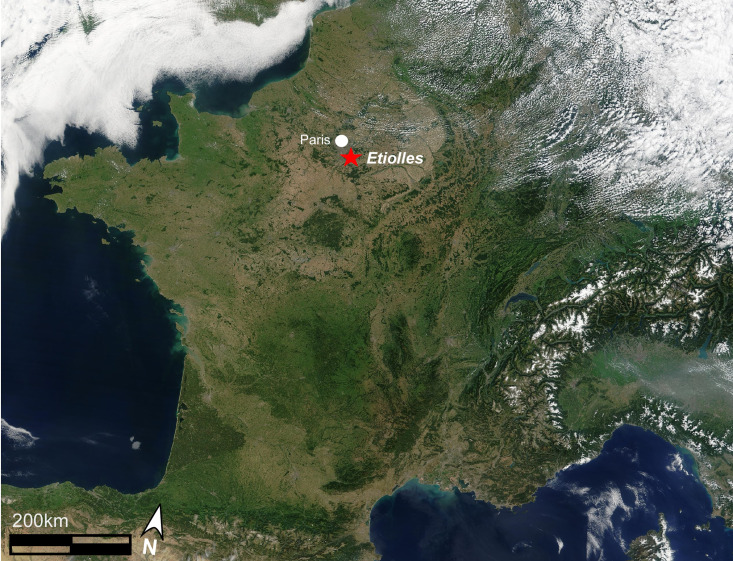
Location of Étiolles, southeast of Paris (NASA satellite image of France in August 2002 by J. Descloitres, MODIS Rapid Response Team, NASA/GSFC).

The landscape of the Paris Basin has retained an important record of Upper Palaeolithic lifeways. Here, a handful of sites retain several exceptionally well-preserved living floors allowing researchers the rare opportunity to study high-quality data pertaining to habitation construction, the use of space within and between tents, and consequently, aspects of social organisation frequently beyond the reach of archaeologists. One of these Paris Basin records is the Late Magdalenian site of Étiolles (Figure 2).

At Étiolles, almost 50 years of excavations and investigations have identified the remains of numerous habitation structures and activity foci revolving around ample – mostly lithic-based – evidence (Olive, 1988, 1992; Olive et al., 2019; Pigeot, 1987a, 2004, 2010). One of the most interesting insights this site has produced is the revelation that the community appears to have had quite strict rules or traditions regarding the use of space within their tents (discussed below). This site has also produced evidence for both master and apprentice flintknappers – and with evidence for apprentice or novice knappers generally accepted to provide strong evidence for the presence of children and/or adolescents on a site (e.g. Bodu et al., 1990; Grimm, 2000; Olive, 1988; Pigeot, 1990, 2010; Shea, 2006), this record provides opportunities to study the activities of both young and old.

**Figure 3.**
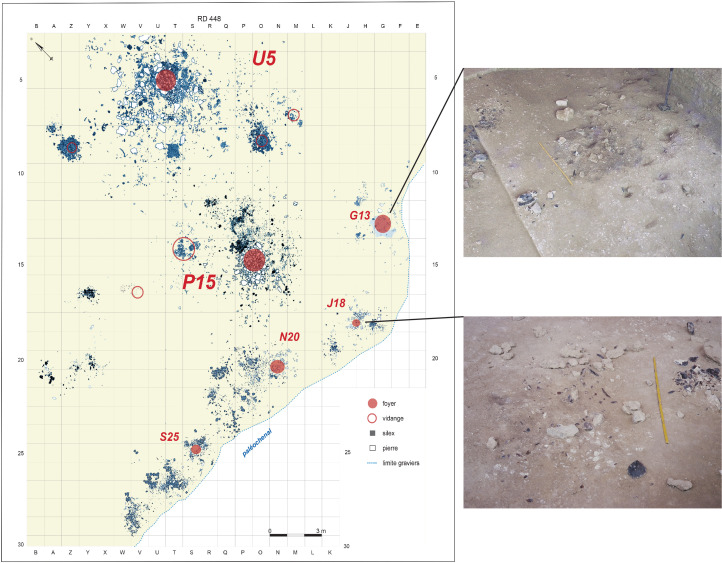
Spatial distribution of archaeological evidence found around the main habitation features U5 and P15 (distribution map courtesy of M. Olive and N. Pigeot, ARPE; Photographs courtesy of ARPE).

Here, I will focus on the U5–P15 level of Étiolles, a living floor located in Locus 1 of the site, and which contains evidence for six archaeological features dated to between 13,160 and 12,800 BP situated next to a stream – the Ru des Hauldres – a tributary of the Seine (Olive et al., 2019; Figure 3). The U5–P15 level has been excavated over a 700 m^2^ area, and is interpreted as part of a campsite consisting of two dwellings (called U5 and P15 respectively) and four associated activity areas that have been given several names, including ‘*foyers d'activaté satellites*’ (Olive, 1992), ‘*foyers secondaires*’ (Pigeot, 2004), and ‘*unités annexes*’ (Olive et al., 2019). Extensive exercises in lithic refitting and analysis of the site's stratigraphic matrix have demonstrated that the six features represent either one extended stay over several months (and a change of season) or two seperate stays interrupted by a short time interval (Olive et al., 2019; Pigeot, 1987b). This two-phase stay is indicated by the fact that the largest shelter – U5 – experienced two main phases of use on the Étiolles river bank. Specifically, the tent was rotated on the spot to change the direction of its openings (presumably in response to changed climate conditions) after a period of extensive use (Olive et al., 2019). Movement of lithic material between U5 and the neighbouring P15 shelter indicates that the latter was only present during the first phase of site use. Similar evidence ties G13, J18, N20, and possibly S25 to the earlier phase. N20 also saw use in the later phase (Olive et al., 2019).

Study of the lithic material making up the U5 and P15 features found that a master knapper was producing high-quality large to very large blades out of U5 that were distributed across the camp site. Blades were also produced in P15, although in much smaller numbers and with much less skill (Olive et al., 2019). Similarly, while the inhabitants of U5 observed strict rules of space use, movements within P15 appear to have been more relaxed, although a division of space between flintknapping and other activities is still apparent. Olive et al. (2019) therefore asked the question: was P15 a place for young people, who were initially confined to a single tent before they were able to move into U5 (thus explaining the disappearance of P15 in the second phase of the site use)? They argue that such an interpretation is not supported by the evidence, which shows that children (indicated by apprentice-produced lithics) were visiting U5 from the start of the stay. Furthermore, distribution of lithic debitage suggests that children were not confined to a particular place, but were instead free to occupy all spaces, as shown by the wide spatial distribution of apprentice-produced debitage. We will return to these issues below.

Significant differences between the large U5 and P15 dwellings and the four smaller features (G13, J18, N20, and S25) have been identified (Olive, 1988, 1992; Olive et al., 2019; Pigeot, 1987a). Specifically, while the features U5 and P15 consist of large concentrations of lithic artefacts surrounding a central, well-built hearth and enclosed an area more than 70 m^2^ in size, the smaller features only cover about 10–30 m^2^ each (Olive, 1992). In addition to their significantly reduced size, Olive (1992) determined that, in contrast to the large ‘*unités d'habitation*’, the four satellite activity areas differed in their location, their composition of artefacts, and their artefact density. These differences led Olive (1992, pp. 122–123) to suggest that the smaller features could be interpreted as ‘functionally versatile locations where multiple tasks have been performed’ (‘*des lieu fonctionnellement polyvalents où ont été effectuées des tâches multiples*’) or phrased another way, as ‘community activity areas’ (‘*zones communautaires*’).

Between the four satellite activity areas (G13, J18, N20, and S25), differences also exist. S25 is distinguished by its relative isolation from the other features. Olive et al. (2019) identified only three links between S25 and the other features: two to neighbouring N20, and one with J18. No evidence for either a covered structured or apprentice knappers was found here, and tasks seem to revolve around the preparation of cores ready for more advanced working elsewhere on site, as well as the processing of game (bison and reindeer) near an unstructured hearth. N20, on the other hand, has the strongest links with the other features and has significantly more remains than the other three small activity areas. Like P15, N20 reflects the strict structured use of space surrounding the hearth, with more intense and more diverse activities undertaken here than in G13 or J18 (Olive et al., 2019). Olive et al. (2019, p. 73) suggest that this feature may reflect ‘an outdoor activity area where more tasks have been performed over a longer period of time by more people than in the smaller units [of G13 and J18]’ (‘*une aire d’activité extérieure où davantage de tâches ont été effectuées sur un temps plus long par davantage de personnes que dans les unités plus petites*’). Along with tool production and repair which were undertaken by those of different skill levels (and presumably, also, ages), evidence for the consumption of meat at N20 is attested to from faunal remains, and the presence of both ochre and finished tools indicates the undertaking of other work, such as hide preparation and decoration. In sum, S25 is suggested to have been the location of butchering prey and reducing cores with N20 being a place for a wide range of activities including tool-making and use, and the preparation and consumption of meals (Olive et al., 2019).

G13 and J18 differ again. These two features are significantly smaller than both S25 and N20, have fewer artefactual remains associated with them, and have the strongest evidence for children in the form of material produced by apprentice flintknappers. Interestingly, Olive (1992, p. 123) states that ‘it seems to us premature to deduce an attribution of this place to particular members of the social group (in this case children)’ (‘*Il nous sombre aloes prématuré d'en déduire une attribution de ce lieu à des membres particuliers du group social (en l'occurrence des enfants)*’). In their later work, however, Olive et al. (2019) are more confident in their site assignations, stating that for G13, ‘this small site appears above all as a place of learning where a beginner knapper started. A modest activity of working (debitage, retouching of a burin) and the repeated use of the hearth suggest the undertaking of tasks related to the fire, perhaps the presence of one or more adults (women?) occupied with culinary and technical work’ (‘*Ce petit ensemble apparaît surtout comme un lieu d’apprentissage où s’est initié un tailleur débutant. Une modeste activité de taille (débitage, réavivage de burin) et le fonctionnement répété du foyer suggèrent l’accomplissement de tâches en rapport avec le feu, peut-être la présence d’un ou de plusieurs adultes (femmes?) occupés à des travaux culinaires et techniques*’) (Olive et al., 2019, p. 161). And of J18, ‘we perceive the presence of knappers of unequal skill, a qualified knapper from U5 and having returned there to continue shaping of a core, and an apprentice who settled near the hearth. This unit clearly participates in the circulation network of people and prepared products: cores arrive there, others leave, blades are produced there, some are removed and transported elsewhere, adults come to exploit the cores there, young practice at the hip’ (‘*On perçoit la présence de tailleurs de compétence inégale, un tailleur qualifié venu de U5 et y étant retourné pour y poursuivre la mise en forme d’un nucléus, et un apprenti qui s’est installé près du foyer. Cette unité participe clairement au réseau de circulation des personnes et des produits de la taille : des nucléus y arrivent, d’autres repartent, des lames y sont produites, certaines sont prélevées et transportées ailleurs, des adultes viennent y exploiter les nucléus, des jeunes s’y exercent à la taille* ’) (Olive et al., 2019, p. 161).

The presence of solid evidence for child actors contributing to the creation of the Étiolles record – and specifically the two discrete features of G13 and J18 in particular – provides an opportunity to ask the question: are these archaeological residues more than just locations where apprentice flintknappers practised? Could they also represent children's playhouses/secret spaces? In order to explore this idea, I will compare the reported archaeological record with the eight characteristics of child-built secret spaces outlined above.

### 1. A location ‘out of sight’ of adults

**Figure 4.**
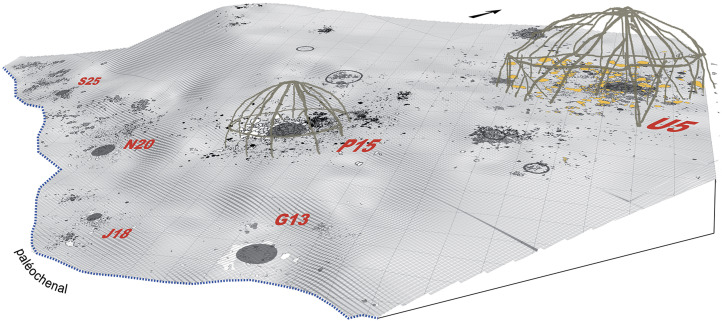
Location of G13 and J18 in comparison with P15 and U5 shelters (after Olive et al. 2019: Fig. 6; drawn by Y. Le Jeune and N. Pigeot).

G13 and J18 were situated along the bank of the small tributary, the Ru des Hauldres. While such a location might be considered advantageous for undertaking some activities (such as particular material culture manufacture or repair), it would also appeal to children trying to carve out a space away from adult eyes. Being down on the stream bank takes advantage of the steeply slopping bank in order to ‘hide’, or at least, be partially obstructed from the view of those in or outside of the large U5 dwelling. With forests or copses of trees somewhat sparse during the Magdalenian period (and therefore possibly quite a distance from where the camp was placed), and taking into account parental restrictions regarding how far away children could wander from camp, the stream bank may have been the only natural shield from view available to the children of Étiolles. Along these lines, it is interesting to note that G13 is also around the bend of the stream, meaning that activities in this location are a little removed from direct sight of those working at N20 and S25 further down stream (Figure 4).

Children's independent mobility – their ability to move around without adult accompaniment – is vital for their physical, social, cognitive and emotional development as it promotes physical activity, peer bonding, independence, and mental health (Hillman et al., 1990; Kyttä, 2004; Prezza & Pacilli, 2007; Prezza et al., 2001). Recent studies have found that the distance children are able to travel from home alone is dependent on their parents’ fears for their safety (Carver et al., 2008; Veitch et al., 2006; Villanueva et al., 2012). The situating of the G13 and J18 features, therefore, could be consistent with children moving as far from their parents (to the edge of the stream) and as out of sight (even only if it is in their own perception) down the stream bank as the camp site allowed.

### 2. Small site size

As mentioned above, there is a significant difference in the overall size of the archaeological features identified as the habitation tents (between 70 and 180 m^2^), the first two of the ‘smaller activity areas’ (that is, S25 and N20 at around 30 m^2^ each), and then G13 and J18, both only reaching 16 m^2^ (Olive, 1992; Olive et al., 2019).

Notable differences also exist in the quantity of lithic artefacts – in terms of both total quantity recovered and number of particular types – which help define each feature as outlined in Table 1. Furthermore, within the overall feature limits of G13 and J18, small spaces were identified and will be described below.

**Table 1.** Number of lithic artefacts in each of the six features of the U5–P15 level of Étiolles (after Olive et al., 2019)

Feature type	Feature	Area	Total lithic artefacts (including hearth stones)	Cores	Finished tools
Dwelling/enclosed tent	U5	180 m^2^	26,738	73 (0.3%)	505 (1.9%)
	P15	70 m^2^	8,128	22 (0.3%)	53 (0.7%)
Outdoor activity area	S25	30 m^2^	2,869	11 (0.4%)	10 (0.3%)
	N20	30 m^2^	2,622	10 (0.4%)	11 (0.4%)
Child secret space?	G13	16 m^2^	791	3 (0.4%)	4 (0.5%)
	J18	16 m^2^	1,152	6 (0.5%)	1 (0.08%)

### 3. Mimicry of the main housing type utilised by the child's community

The identification of shelters at Étiolles is based on the presence of large stones encircling a central well-built hearth surrounded by a dense crown of artefactual remains, and several clusters of lithic remains (dumps) outside the dwelling (Coudret et al., 1994; Masson, 1982; Olive, 1988; Pigeot, 1987a; Rieu, 1985). The U5 dwelling appears to have had two exits: one towards the south (towards the steam) and the other towards the west. A third towards the north is also a possibility (Olive et al., 2019).

Because living shelters house work related to everyday needs, but presumably also more occasional activities such as beading and mobile art production, the types of artefacts recovered in such places are diverse and will sometimes include non-utilitarian objects such as ornaments. Free space to work is necessary, and such ‘empty’ places are found amongst the debris left around the hearth, but within the shelter confines. A strict use of space within U5 (and other large enclosed structures) at Étiolles was alluded to above. This information was drawn from analysis of the lithic evidence and found that the inhabitants organised the use of covered space according to three categories associated with flintknapping skill: novice knappers were relegated to the periphery of the enclosed space, adept knappers closer to the central hearth, and master knappers most central of all. Olive et al. (2019) found that these rules are not as strictly followed outside of the U5 tent, including when in the neighbouring P15 tent, which while not as clear in its presentation as an enclosed space, still presents enough indices for the existence of a covered tent to also be supported. Differences between U5 and P15 are such that the excavators propose that this latter structure was ‘subordinate’ to what appears to be the main house (U5) (Olive et al., 2019).

Activities carried out inside U5 include the repairing of hunting weaponry and the working of bone and hide, as well as the preparation and consumption of meals. Ochre is present, as is a small number of tooth ornaments, suggesting the manufacture or maintenance of decorated clothing (or similar) (Olive et al., 2019). P15 has a comparable diversity of manufacturing and food activities, although artefact deposition is less dense and the quality of the lithic tools produced is not as polished as that presented in U5 (leading to the suggestion that younger people inhabited this tent).

In comparison, S25, N20, G13, and J18 are all argued by Olive et al. (2019) to have been outdoor activity areas, although Olive (1992, p. 122) states in her earlier analysis that a ‘light protective structure cannot be ruled out’ for G13 and J18. Both G13 and J18 feature hearths, although they are about half the size of those maintained in U5 and P15 (which are around 100 cm in diameter). That of G13 is described as ‘a flat hearth with a few stones that form an incomplete circle about forty centimetres in diameter’ (‘*un foyer plat aménagé à l'aide de quelques pierres qui dessinent un cercle incomplete d'une quarantaine de centimètres de diametère*’) (Olive, 1992, p. 95). The hearth stones are almost exclusively sandstone – the material favoured for this purpose in the larger structures. The biggest difference in the choice of hearthstones is their size. In G13, the stones are only around 10 cm long as compared with the much larger ‘slabs’ laid down in U5 (Olive, 1992). The hearth itself saw repeated and prolonged use. Most of the blade blanks and the rare preserved bones fall on the south side of this hearth, with the opposite side (north and west) characterised by two concentrations of lithics.

For J18, the hearth stones have been scattered so that their original layout is no longer visible, although its presence is indicated by blackened sediment spread over about 60 cm in diameter. The scattered hearth stones are similar to those found in G13, measuring around 10 cm in diameter. The fire here appears to have been either little used or very carefully curated with the contents of the hearth dumped towards the east – a behaviour witnessed for U5 (Olive, 1992; Olive et al., 2019). In contrast, the collection of lithic debitage from around the hearth and its dumping away from the activity foci is not seen in either G13 or J18, despite this behaviour being clearly observed for the large dwellings (Olive et al., 2019). The separation of southern and northern activity areas (as in G13) is less distinct here, mostly owing to the domination of lithic production at J18 (with at least 12 operations having been carried out at this location).

Other behaviours regarding the use of space observed for the tents of U5 and P15 include the working of flint on one side of the hearth and food preparation on the other. Olive (1992) concluded that this pattern of activity ‘was presumably a traditional component of Magdalenian lifeways’ (Olive, 1992, p. 101), and we see this same spatial separation of flintknapping from food preparation reflected in both G13 and J18 (discussed more below).

### 4. Ephemeral in nature

As noted, the lithic material found at G13 and J18 is limited in comparison with that recovered within and immediately surrounding the larger housing units (Table 1). Similarly, evidence for fire use is more ephemeral at G13 and J18, with ochre (found as stained sediments in U5 and P15) and ornaments completely absent from both G13 and J18 (Olive, 1992). In absolute measures, the overall size of features G13 and J18 and their artefactual composition are far more ephemeral than the significant U5 and P15 structures. Indeed, these smaller features could easily have been erased by weather events (at least) being situated at the edge of the Ru des Hauldres stream and it is only the extraordinary preservation of the Étiolles site that has led to their discovery some 13,000 years later.

### 5. A structure may be present – but not necessarily (age dependant)

Neither G13 nor J18 preserves strong evidence for a structure having been constructed over the hearths, with both lacking the clear circle of stones that supported a tent like that seen for U5 and P15. Despite this fact, Olive (1992, p. 122) states that a ‘light protection structure cannot be ruled out’ and the above noted similarities in the lay out of activities around the hearth at these small features follows that laid out in the main enclosed structures. Thus, whether a shelter was present or not, those creating features G13 and J18 were following the spatial rules enacted within the enclosed U5 dwelling.

We should also remember that the construction of a structure in child-built playhouses – and its robustness – is dependant on the age of the children. Sobel (1993, pp. 128–129) reports, for example, that ‘huts ranged from sticks arranged on the ground by the youngest children to constructed, enclosed huts by the 7- and 8-year-olds to elevated, multilevel tree forts by the “biggies” [children aged up to 12-years-old]’ in a study of a primary school setting located in Melbourne, Australia. He also observed this age-differentiated style of playhouse building in other, very different, cultural settings, suggesting that this behaviour is universal in children. His additional observation that, after the age of 8, girls become more focused on the interior details of their spaces while boys concentrated more on the built structure itself should also be kept in mind – for, if the children at Étiolles (and we know there were children) were building playhouses, the form that these play spaces took for the seasons that the U5–P15 level was inhabited would vary according to the age of the children present. Consequently, if the groups of children were under the age of eight, we could not expect a substantial structure to have been constructed. In any case, the vagaries of archaeological preservation would not be kind to recording even more solidly build child-produced shelters.

### 6. Low-value items and refuse

The hostile organic preservation conditions of the Étiolles site severely hampers our ability to identify the use of non-lithic materials, even more durable ones, such as antler and tooth. Hence, we are left only with the lithic materials to investigate the use of refuse or other low-value items that may have been given or otherwise collected by children.

In both G13 and J18, cores which have become less productive as well as large flakes have been taken up by unskilled flintknappers and further worked (Olive, 1992; Olive et al., 2019). Refitting has determined that these cores and flakes were picked up in the larger structures (either U5 or P15) and taken to G13 or J18 (Olive et al., 2019). Similarly, the long blades present in the small units were not produced on site, but instead by the expert knapper/s within U5. In fact, while a certain level of reciprocity in blades is identified between U5 and P15 (although with many more blades moving from the former to the later and of higher quality), all connections with G13 and J18 are one way: from the large dwellings to the small features (Olive et al., 2019). This direction of movement is consistent with the behaviour of children either being given or otherwise collecting low-value items from adult places for their own purposes (see Baxter, 2005b).

Also notable is the fact that high-value items are absent from both G13 and J18, specifically ochre, weapon tips, and ornaments, as are finished typologically ‘classic’ tools such as scrapers and burins (Olive, 1992). Similarly, while retouched tools (especially backed blades) are common within U5 (*n =* 505) there are very few within G13 (*n =* 4) and J18 (*n =* 0) (Olive et al., 2019).

### 7. Children's material culture

Items that are clearly children's playthings are thus far absent from Étiolles; however, several pieces of *art mobilier* (including a flaked stone horse figurine and a stone resembling a female form) may constitute such material culture (Pigeot, 2004). Despite this situation, the strongest indicators of the presence of children at G13 and J18 is the extensive evidence for apprentice flintknappers (Morgenstern, 1990; Olive, 1992; Olive et al., 2019; Pigeot et al., 1991). At both locations (and elsewhere at Étiolles), cores show poor control of gestures during percussion and the inability to rectify accidents (Olive, 1992).

At G13, apprentice knappers reduced four cores in the northwest corner of the feature, with three of the cores showing clear signs of awkward handling and which ‘have such close technological characteristics that is permissible to ask whether they are not the work of the same individual’ (‘*les trois débitages possèdent des caractéristiques technologies si proches qu'il est permis de se demander s'ils ne sont pas l'oeuvre du même individu*’) (Olive, 1992, p. 106). Indeed, one of these cores was large enough to produce blades around 10 cm long; however, the knapper only achieved examples 7–8 cm long with most just 5 cm in length owing to lack of platform preparation and poor gesture control (Olive, 1992). Also found here is a small accumulation of spalls from the resharpening of a burin, although the tool itself is absent. Items prepared in this northwest corner of G13 were brought to the hearth for use, though the nine blades found around the hearth were not made on site, but in P15. No cores or blanks moved from G13 back to P15 (Olive et al., 2019). Olive et al. (2019) suggests that the lithic evidence for G13 therefore reflects one or two apprentice flintknappers having used the location to practice their skills, and in general, that G13 presents as a low-traffic but versatile location where activities related to lighting/heating and maintenance of a home predominate.

At J18, more intense flintknapping activity is recorded. Debitage suggests at least two knappers of unequal skill: an apprentice discarding awkwardly produced lithics and an experienced knapper preparing a core that produced a significant number of blanks later used in the U5 dwelling (a teaching moment?) (Olive et al., 2019). Knapping is not as spatially restricted here as it was at G13, with shaping occurring at the hearth as well as at a location just to the west. As at G13, a number of blades (20 in this case) are left around the hearth. Movement of knapped material to or from J18 is restricted to one case where a core was poorly worked in J18, briefly passing through G13, before being finally discarded in N20 (Olive et al., 2019). Also found in J18 are two small naturally fractured river cobbles. These 10–14 cm long cobbles were subject to only a few strokes of percussion and were recovered either near to the camp or on the banks/bottom of the stream (Olive, 1992). What behaviour these cobbles represent is unknown. In sum, J18 appears to have been busier than G13, with knapping being the dominant activity, although the preparing and/or cooking of meals also occurred.

In a rare absence of evidence case, an empty space in square H18 (part of feature J18) may indicate the presence of a skin blanket (or similar) laid down next to the hearth on one side and the flintknapping area on the other (Olive, 1992). Similarly, while debitage from the retouching of stone tools (such as burins for organic materials working) is present, the finished tools themselves are largely missing from both G13 and J18 (Olive, 1992; Olive et al., 2019). Indeed, in G13 only two retouched blades and two retouched flakes were found.

Finally, we could also consider the smaller – almost half-size – hearths as possible children's material culture. While smaller does not equate to child, a point frequently pointed out in the childhood literature (e.g. Crawford, 2009; Sofaer Derevenski, 2000), it seems justifiable that children would not produce a fire as large or intense as those kept in the U5 and P15 dwellings.

### 8. Food refuse

Being an open site, faunal remains are exceptionally sparse at Étiolles and consequently it is difficult to determine if food was brought to either G13 or J18. Having said this, blades were found gathered around the hearth in an area where a few remains of bones (three fragments including a bovine rib and perhaps some reindeer) were found in G13, while in J18, blades are again clustered around the hearth and two indeterminate bone splinters were recovered. In both cases, it is suggested these remains indicate the preparation and possible consumption of meals at both locations by the excavators (Olive, 1992; Olive et al., 2019).

## Discussion

While being limited to a reexamination of the published data of Étiolles, it is clear that the features G13 and J18 display evidence for all of the eight characteristics here proposed for identifying children's places. Each feature was located downslope from the household structures (out of sight), small in both absolute and relative terms, ephemeral in nature, and contained evidence for the following of cultural habits regarding the use of space, ample evidence for the activities of apprentice flintknappers and possible evidence for food preparation and/or cooking. Here then, I would argue, is a strong case for the archaeological recording of children's secret spaces in a deep time context. Based on the attention to detail in the spatial organisation of activities, it might further be suggested that the apprentices knapping – and therefore the children creating – these spaces were between 6 and about 8–11 years of age.

Olive et al. (2019) found that evidence for children's activity (knapping) was only absent from the last feature located along the stream (S25). Otherwise, children appear to have not been restricted in their use of the Étiolles camp site, and were practising flintknapping in the company of competent adults at a number of locations. In this context, G13 stands out as a particularly child-produced/utilised site, owing to the density of apprentice-knapped materials. If we reinterpret the two small features – G13 and J18 – as child-built ‘secret spaces’ then the U5–P15 level of Étiolles has recorded not only the work and traditions of a late Magdalenian group going about their day-to-day lives, but also modern-patterned play in their children.

**Figure 5.**
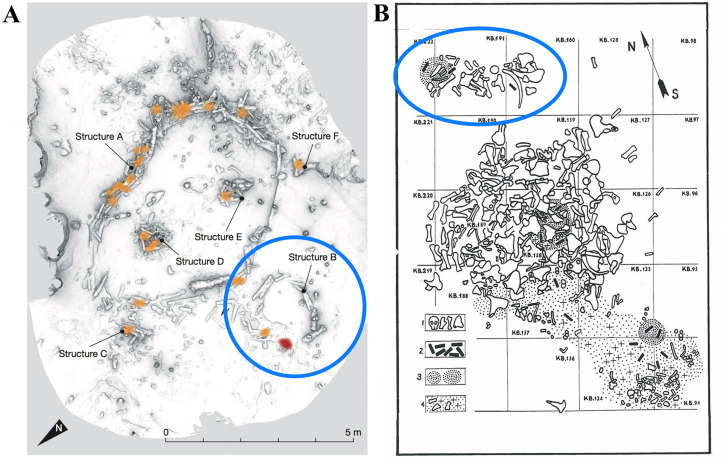
Small activity areas (highlighted by blue circle) elsewhere – children's secret spaces? The Neanderthal site of Bruniquel, France dated to c. 176,500 BP (After Jaubert et al., 2016: Fig. 1) – two circles were made using stalagmites – and the Modern Human EpiGravettian site of Mezhirich, Ukraine (after Pidoplichko, 1998: Fig 26). Here, a small hearth and concentration of bones sit behind a large mammoth bone hut.

A brief review of the Palaeolithic literature finds two examples worthy of further consideration in the future. The first was made by a Middle Palaeolithic Neanderthal community some 176,500 years BP (Figure 5a). In southwest France, two circular structures made from whole and broken stalagmites were discovered inside Bruniquel cave (Jaubert et al., 2016). A small 2.2–2.1 m circular structure (structure B) was found next to a much larger (6.7–4.5 m) oval-shaped structure (structure A). At the entrance of the smaller structure B is evidence for heating and burnt bone remains, while evidence for heating is found in several locations within structure A.

The second example is found at the Ukrainian EpiGravettian site of Mezhirich (Figure 5b). This Upper Palaeolithic site is famous for the preservation of mammoth bone huts (Pidoplichko, 1998), and interestingly, behind the third dwelling were ‘two small reserves of mammoth bones’ (Pidoplichko, 1998, p. 103). These bones were placed on the surface (as determined by the degree of weathering) in a pile some 2.2 m long by 1 m wide and in a natural hollow situated on a slope moving away from the main huts. A subsequent excavation found a second reserve of bones some 1.5 meters to the northwest of the same dwelling and also situated in the hollow. This one is reported to be ‘oval in plan, compact, 1.6 × 1/3 metres in size’ and ‘around the second reserve were some bone fragments (many burnt), sooty traces, pieces of ochre and amber, flint flakes and a few tools (burins), making up a cultural layer about 5 cm thick’ (Pidoplichko, 1998, p. 104).

In both of these cases, the small feature is situated where it cannot be seen by individuals residing inside the larger structure (directly next to the circle at Bruniquel and behind the hut at Mezhirich). They are also comparatively small – and may be mimicking the main structures at each site using unwanted or discarded materials. The similarities of these small features in terms of location (down slopes/hollows or otherwise out of sight from the habitation structures) and character of the record (small, possible use of discarded materials, some tools present, small hearths, and few burnt bones) suggest that a careful reconsideration of what these features represent – and if they may be children's places – is justified. If they can be confidently linked to children's activities, then the demonstration of Neanderthal children following a modern pattern of play is a significant finding in itself.

Indeed, numerous new avenues of thought and research are opened up within Pleistocene archaeology if we are able to identify children's places. In modern times, children within hunter–gatherer communities are commonly less restricted than those in urban societies, with independence emphasised and interaction with age-mates making up a substantial portion of their daily routine. Yet how similar is this modern scenario to that experienced by hunter–gatherer children many thousands of years ago? Did they also have freedom to roam far from their guardians or were they more constrained at different times throughout the past? Did they always have access to age-mates or only at certain times? What impact did these circumstances have on the cognitive and behavioural development of children? Is the impulse to build cubbies and play ‘house’ with your siblings, cousins, and/or friends related to an evolutionary mechanism for establishing cooperation within a group? How different was the play behaviour of early *Homo sapiens* with other human species? These are but a very few questions which archaeologists can now explore.

## Conclusion

To conclude, I want to bring us back to the recent sociological and psychological literature which reminds us that children regularly express ‘a need for privacy, independence, and self-sufficiency. Through making their own places, children start to carve out a place for themselves in the world’ (Sobel, 1990, p. 47). We can be sure that such needs were also present in past Modern Human children, and therefore that the residues of this universal behaviour are preserved in some way in at least some deep time contexts. The extraordinary preservation conditions of the Magdalenian site of Étiolles provides an excellent test case, although further development of the methodology presented here may allow the identification of children's spaces in less well-preserved sites – and this is the goal we should next set our sights on.

## Data Availability

All data discussed is presented here in or in the cited references.
